# Baseline cerebral oximetry values in cardiac and vascular surgery patients: a prospective observational study

**DOI:** 10.1186/1749-8090-5-41

**Published:** 2010-05-24

**Authors:** Nikolaos G Baikoussis, Menelaos Karanikolas, Stavros Siminelakis, Miltiadis Matsagas, Georgios Papadopoulos

**Affiliations:** 1 Department of Cardiac Surgery, University of Ioannina School of Medicine, Stavrou Niarchou Avenue, Ioannina 45110, Greece; 2 Department of Anaesthesiology and Critical Care Medicine, University of Patras School of Medicine, Rion 26500, Greece; 3 Department of Vascular Surgery, University of Ioannina School of Medicine, Stavrou Niarchou Avenue, Ioannina 45110, Greece; 4 Department of Anaesthesiology and Postoperative Intensive Care, University of Ioannina School of Medicine, Stavrou Niarchou Avenue, Ioannina 45110, Greece

## Abstract

**Aim:**

This study was conducted to evaluate baseline INVOS values and identify factors influencing preoperative baseline INVOS values in carotid endarterectomy and cardiac surgery patients.

**Methods:**

This is a prospective observational study on 157 patients (100 cardiac surgery patients, 57 carotid endarterectomy patients). Data were collected on factors potentially related to baseline INVOS values. Data were analyzed with student's t-test, Chi-square, Pearson's correlation or Linear Regression as appropriate.

**Results:**

100 cardiac surgery patients and 57 carotid surgery patients enrolled. Compared to cardiac surgery, carotid endarterectomy patients were older (71.05 ± 8.69 vs. 65.72 ± 11.04, P < 0.001), with higher baseline INVOS (P < 0.007) and greater stroke frequency (P < 0.002). Diabetes and high cholesterol were more common in cardiac surgery patients. Right side INVOS values were strongly correlated with left-side values in carotid (r = 0.772, P < 0.0001) and cardiac surgery patients (r = 0.697, P < 0.0001). Diabetes and high cholesterol were associated with significantly (P < 0.001) lower INVOS and smoking was associated with higher INVOS values in carotid, but not in cardiac surgery patients. Age, sex, CVA history, Hypertension, CAD, Asthma, carotid stenosis side and surgery side were not related to INVOS. Multivariate analysis showed that diabetes is strongly associated with lower baseline INVOS values bilaterally (P < 0.001) and explained 36.4% of observed baseline INVOS variability in carotid (but not cardiac) surgery.

**Conclusion:**

Compared to cardiac surgery, carotid endarterectomy patients are older, with higher baseline INVOS values and greater stroke frequency. Diabetes and high cholesterol are associated with lower baseline INVOS values in carotid surgery. Right and left side INVOS values are strongly correlated in both patient groups.

## Introduction

Persistent cognitive decline or permanent neurologic deficits are common after cardiac or vascular surgery [1]. A large prospective study reported that serious neurological deficits occur in up to 6.2% of patients after myocardial re-vascularization [2], and factors other than emboli seem to be involved in more than 50% of cases. A study by Slater et al [3] showed that the incidence of early postoperative cognitive decline was 60%. Other data show that more than 40% of patients undergoing cardiac surgery develop persistent cognitive decline resulting in functional impairment [4] and prolonged hospital stay [3], and, according to current thinking, embolism is not the sole cause of these phenomena. Cerebral oximetry, as measured by INVOS, is a promising neuro-monitoring technology[5], but its usefulness during cardiac surgery, vascular surgery, and in the cardiovascular ICU has not, as of yet, been adequately evaluated.

Non-invasive cerebral oximetry uses near-infrared reflectance spectroscopy (NIRS) to measure frontal lobe regional cortical oxygen saturation. Measurement is based on the different absorption characteristics of oxygenated and deoxygenated hemoglobin: oxygenated hemoglobin (HbO_2_) absorbs less red light (600-750 nm) and more infrared light (850-1000 nm) than deoxygenated hemoglobin. As a result, deoxygenated hemoglobin has an absorption peak at 740 nm while HbO_2 _does not [5]. Consequently, the fraction of oxyhemoglobin can be determined by using two infrared wavelengths, thereby providing a technique for continuous non-invasive, bed-side monitoring that reflects the balance between cerebral oxygen supply and demand [5]. Other techniques, such as jugular venous saturation and electroencephalography have also been used [6], but in this study we only evaluated INVOS.

An association between cerebral oxygen desaturation during cardiac surgery and postoperative cognitive dysfunction, prolonged intensive care unit (ICU), and hospital stay has been demonstrated [7], and intraoperative cerebral ischemia and cerebral oxygen desaturation have been proposed as possible mechanisms contributing to postoperative cognitive dysfunction [7,8]. In addition, a RCT conducted by Murkin and colleagues [9] demonstrated that treatment of cerebral oxygen desaturation was associated with shorter ICU length of stay, significantly reduced incidence of major organ morbidity, and lower mortality. Cerebral oximetry monitoring is increasingly used to monitor frontal lobe perfusion during cardiac and non-cardiac surgery. Furthermore, the use of INVOS has been reported to help detect aortic cannula displacement, and some authors have suggested that all cardiac surgery patients should have intraoperative cerebral oxygenation monitoring [10].

Perioperative stroke is an inherent risk of carotid endarterectomy and occurs in 5-7.5% of patients [11]. As hypoperfusion during cross clamping is a major cause of stroke, CEA can be considered as a human model of regional cerebral ischemia, and may provide an ideal opportunity for evaluating the role of INVOS as a monitor of cerebral ischemia.

Not surprisingly, cerebral oximetry has been used in several investigations on patients undergoing CEA [5], and there is significant correlation between carotid stump pressure and cerebral oximetry during carotid endarterectomy [12]. In the last decade, technological research has expanded the application of NIRS to allow continuous, non-invasive bed-side monitoring of cerebral tissue oxygen saturation through the scalp and skull, thereby providing accurate useful information on the balance between brain oxygen supply and demand [5]. Due to the variability of baseline rSO_2 _values between patients, a baseline should be determined for each patient before induction of general anesthesia, and detection of cerebral ischemia is based on deviations from baseline, rather than on absolute INVOS values. Generally, a 20% reduction below baseline is considered evidence of cerebral ischemia [13,14]. However, if baseline rSO_2_is < 50%, then reduction by 15% below baseline is the critical threshold for ischemia detection. Data suggest that routine use of rSO_2 _monitoring to guide the anesthesia plan during cardiac surgery may improve patient outcome and shorten hospital stay [5,11,15]. Several studies have attempted to define the risk factors and the conditions influencing rSO_2 _baseline, and age is considered the strongest predictive factor for postoperative cognitive dysfunction (POCD) after cardiac surgery [16]. In addition to advanced age, other reported risk factors for POCD after coronary artery bypass graft surgery (CABG) are systemic inflammation[17], low education level, diabetes, severity of atherosclerotic disease and type of surgery [1,16].

This study was conducted to determine factors associated with preoperative baseline INVOS values in patients undergoing CABG, valve replacement or carotid endarterectomy surgery. Hematocrit, sex, anthropometric characteristics, blood oxygenation, cerebral blood flow, cerebral metabolic rate and head position can influence rSO_2 _[5]. Hypocarbia, and inadequate mean arterial pressure (MAP) are additional factors influencing rSO_2 _[18]. In this study we attempted to evaluate the relationship, if any, of other variables, such as left ventricle ejection fraction, side of carotid stenosis, history of cardiac ischemic and/or cerebrovascular event on baseline preoperative INVOS values.

## Methods

This prospective, non-randomized, observational study was conducted at the University Hospital of Ioannina between October 2007 and December 2008. The study was approved by the Institution Ethics Committee, and all patients gave written informed consent for data collection. 100 patients undergoing cardiac surgery and 57 patients undergoing carotid surgery enrolled.

**Inclusion criteria were **elective carotid or cardiac surgery and age > 18.

**Exclusion criteria were: **emergency surgery, surgery starting after 18.00, age > 90, renal failure requiring hemodialysis, advanced liver cirrhosis with elevated baseline bilirubin or prolonged PT, known dementia and known serious psychiatric disease.

Fifty seven patients scheduled for elective carotid endarterectomy, and 100 patients scheduled for elective cardiac surgery with or without cardiopulmonary bypass (CPB) enrolled. All carotid endarterectomy operations were performed by the same vascular surgeon (MM) without using a shunt. Likewise, all cardiac operations were performed by the same cardiac surgeon (SS). Among patients undergoing cardiac surgery (n = 100), 78 patients had CABG (42 patients with CPB, 36 patients without CPB) and 22 patients had valve replacement surgery.

Demographic data and data on risk factors known or believed to be associated with coronary artery and/or peripheral vascular disease (Age, Gender, Diabetes Mellitus, History of Stroke, Smoking, High cholesterol, Hypertension) were prospectively collected from all patients. Right and Left side baseline INVOS data were recorded before oxygen administration started and before any sedation was given.

### Data collection and analysis

As this is an observational study, we did not conduct any power analysis for sample size estimation, and there was no randomization or blinding. Data were prospectively collected and securely stored in an electronic database.

All data analysis was done with the SPSS v. 16 statistical software package (SPSS Inc, Chicago, IL). Data normality was assessed with the Kolmogorov Smirnov test. Depending on data distribution, continuous data were compared with two-sided Student's t test or the Mann-Whitney U test. Correlations between variables with continuous data were assessed with Pearson's r, and comparisons between proportions were done with Chi-square test. P < 0.05 was considered significant for all comparisons. Linear regression was used to analyze the relative contribution of different variables to observed baseline INVOS variability. The "Statistica" version 7 Statistical Software Package (StatSoft Inc, Tulsa, Oklahoma, USA) was used to generate scatter plots for significant correlations between variables.

## Results

A total of 157 patients enrolled; 100 of those had cardiac surgery and 57 had carotid surgery. Demographic data, risk factors and baseline preoperative INVOS values are presented in Table 1. Patients undergoing carotid surgery were significantly older, and had higher baseline INVOS values and greater frequency of stroke. Diabetes and high cholesterol were significantly more common among cardiac surgery patients (Table 1).

**Table 1:** Demographic data and data on risk factors for coronary and/or peripheral vascular disease in cardiac and vascular surgery patients.

	Cardiac (n = 100)	Vascular (n = 57)	P
Male/Female	70/30	46/11	NS
Age	65.72 ± 11.04	71.05 ± 8.69	0.001
Smoking	65	41	NS
Diabetes	37	13	0.066
High Cholesterol	53	14	0.001
HTN	81	29	0.0001
History of CVA	8	15	0.002
Baseline INVOS Left side	63.25 ± 7.28	66.81 ± 8.17	0.007
Baseline INVOS Right side	62.25 ± 8.04	65.91 ± 8.06	0.007

Results are reported as mean ± SD

### INVOS in vascular surgery

**Figure 1:**
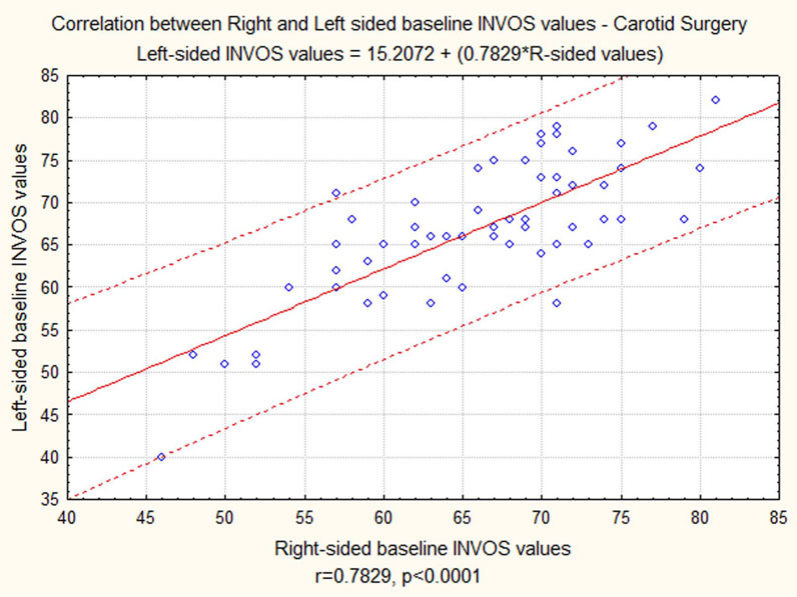
**Graphic presentation of correlation between Right and Left-sided baseline INVOS Values in carotid surgery patients**.

Baseline INVOS values in vascular surgery patients had normal distribution bilaterally. Comparison between the right-sided (Table 2) and left-sided (Table 3) baseline INVOS values with paired t-test showed that there was no significant difference between Right and Left-sided baseline INVOS values. Correlation between right and left-sided baseline INVOS values was evaluated with Pearson's r; this analysis showed that the right and left sided INVOS values are very strongly correlated (r = 0.7829, P < 0.0001). Figure 1 shows graphically the correlation between right and left INVOS values.

**Table 2:** Right side baseline INVOS data in the presence and absence of risk factors in vascular surgery patients

Risk factor	Present	Absent	P
Male sex	66.74 ± 7.92	62.45 ± 8.10	NS
Diabetes	57.00 ± 6.90	68.55 ± 6.34	0.000
Smoking	67.15 ± 7.37	62.75 ± 9.12	0.064
Cholesterol	60.14 ± 8.81	67.79 ± 6.92	0.001
Hypertension	63.52 ± 8.63	68.39 ± 6.71	0.021
CAD	63.00 ± 6.43	66.53 ± 8.29	NS
Asthma	68.25 ± 5.91	65.74 ± 8.22	NS
CVA	68.33 ± 5.97	65.05 ± 8.58	NS

**Table 3:** Left sided baseline INVOS data in the presence and absence of risk factors in vascular surgery patients

Risk factor	Present	Absent	P
Male sex	67.59 ± 7.52	63.55 ± 10.26	NS
Diabetes	60.08 ± 9.03	68.80 ± 6.82	0.000
Smoking	68.20 ± 7.03	63.25 ± 9.94	0.039
Cholesterol	62.86 ± 10.98	68.09 ± 6.69	0.036
Hypertension	66.38 ± 10.04	67.25 ± 5.80	0.691
CAD	66.80 ± 5.12	66.81 ± 8.73	NS
Asthma	67.00 ± 3.56	66.79 ± 8.44	NS
CVA	67.33 ± 5.96	66.62 ± 8.88	NS

Diabetes, smoking and high cholesterol were associated with cerebral oximetry: baseline INVOS values were significantly lower bilaterally in patients with DM (60.08 ± 9.03 on the left, 57.00 ± 6.90 on the right) compared to patients who did not have DM (68.80 ± 6.82 on the left, 68.55 ± 6.34 on the right, P < 0.000). Baseline INVOS values were also related to smoking, with smokers having higher INVOS values on the left (68.20 ± 7.03 vs. 63.25 ± 9.94 in non-smokers, P < 0.039). Age, sex, history of CVA, Hypertension, Presence of CAD, Presence of Asthma, Side of carotid stenosis and Side of carotid surgery (Table 4) were not related to INVOS values. The relationship between the above variables and baseline INVOS values was evaluated with Multivariate analysis, which also showed that Diabetes is significantly associated with lower baseline INVOS values bilaterally (P < 0.001). The presence of diabetes explained 16.5% (p < 0.004) of the observed baseline INVOS variability on the left side, and 36.4% (p < 0.000) of the observed variability on the right side.

**Table 4:** Baseline INVOS values and side of scheduled carotid surgery

	Side of Surgery		
**Baseline INVOS**	**Left surgery**	**Right surgery S**	**P**
Left baseline INVOS	67.96 ± 7.30	65.91 ± 8.80	NS
Right baseline INVO	66.24 ± 6.85	65.66 ± 8.99	NS

Overall, analysis of baseline INVOS data in carotid surgery patients reveals that right and left-side baseline INVOS values are strongly associated with diabetes. In addition, right-side baseline INVOS is associated with high cholesterol and hypertension, and there is also a marginal relationship with smoking (p < 0.064). Left-side baseline INVOS values are associated with smoking and high cholesterol, but not with hypertension.

### INVOS in cardiac surgery

**Figure 2:**
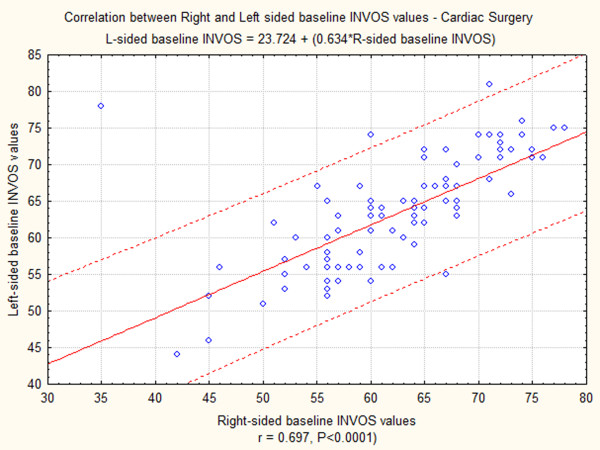
**Correlation between right and left-sided baseline INVOS values in cardiac surgery**. Pearson correlation r = 0.695, P = 0.000.

Baseline INVOS data had normal distribution in cardiac surgery patients. Comparison between right and left-sided baseline INVOS values with paired t-test showed that there was no significant difference between Right and Left-sided INVOS values. Correlation between right and left-sided baseline INVOS values was evaluated with Pearson's r, and showed that INVOS values on the right side (Table 5) and left side (Table 6) are strongly correlated (r = 0.697, P < 0.0001). Correlation between right and left INVOS values is shown graphically in Figure 2.

**Table 5:** Right sided baseline INVOS data in the presence and absence of cardiovascular risk factors in cardiac surgery

Risk factor	Yes	No	P
Male sex	62.49 ± 8.42	61.70 ± 7.19	NS
Diabetes	63.46 ± 6.79	61.54 ± 8.67	NS
CVD	56.75 ± 9.45	62.73 ± 7.79	NS
PVD	60.56 ± 9.28	63.20 ± 7.16	NS
Smoking	62.03 ± 8.56	62.66 ± 7.10	NS
Cholesterol	62.42 ± 8.63	62.06 ± 7.43	NS
Hypertension	62.27 ± 8.42	62.16 ± 6.41	NS
old MI	64.33 ± 6.63	61.70 ± 8.33	NS
Valve Surgery	58.41 ± 10.11	63.34 ± 7.47	0.027

**Table 6:** Left sided baseline INVOS data in the presence and absence of cardiovascular risk factors in cardiac surgery

Risk factor	Yes	No	P
Male sex	63.30 ± 7.47	63.13 ± 6.94	NS
Diabetes	63.38 ± 6.52	63.17 ± 7.74	NS
CVD	62.50 ± 8.72	63.32 ± 7.19	NS
PVD	62.44 ± 7.67	63.70 ± 7.07	NS
Smoking	62.71 ± 6.97	64.26 ± 7.83	NS
Cholesterol	63.49 ± 6.93	62.98 ± 7.72	NS
Hypertension	63.25 ± 7.45	63.26 ± 6.66	NS
old MI	64.29 ± 5.97	62.97 ± 7.60	NS
Valve surgery	61.88 ± 8.56	63.96 ± 7.24	NS

In contrast to our findings in carotid surgery patients, diabetes, smoking and high cholesterol were not associated with baseline cerebral oximetry values in cardiac surgery patients. Age, gender, history of old MI, Hypertension, and the type of operation (valve replacement vs. CABG) were not related to baseline INVOS values on either side.

**Figure 3:**
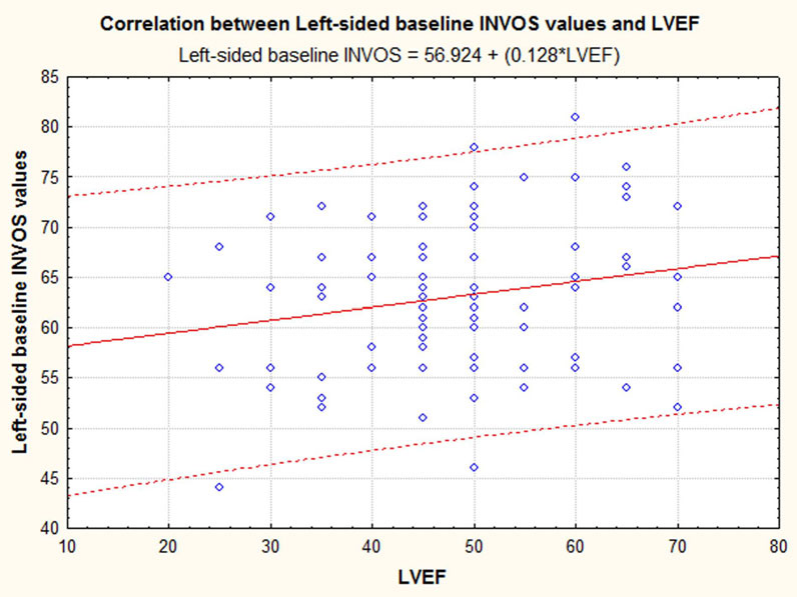
Positive correlation between LVEF and Baseline L-side INVOS values (r = 0.206, P < 0.043)

**Figure 4:**
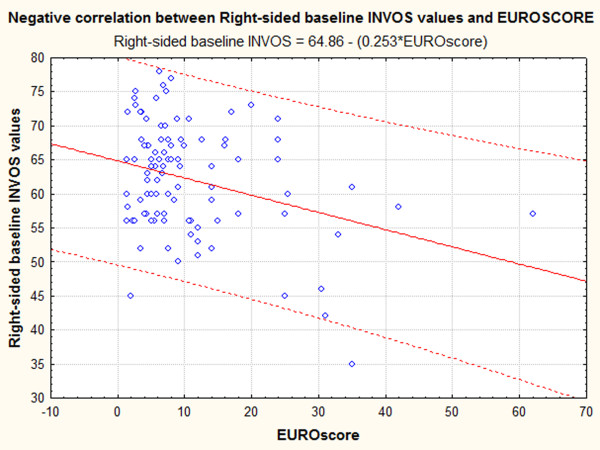
**Correlation between Euroscore and Baseline R-side INVOS values**. Correlation is negative (r = -0.315, P < 0.001).

Linear regression analysis was used to search for variables that could predict right or left-sided baseline INVOS values. Regression was done on 92 cases (8 cases contained missing values), and showed that LVEF and baseline right-side baseline INVOS values are independent, significant predictors of left-side INVOS values. In addition to regression, we also looked for correlations between baseline R or L side INVOS values and weight, height, LVEF and Euroscore. This analysis showed that L-sided INVOS is marginally correlated with body weight (r = 0.192, p < 0.061) and significantly correlated with LVEF (r = 0.206, p < 0.043, Figure 3), whereas the correlation between L-sided INVOS and Euroscore was negative, but did not reach statistical significance (P = 0.09). In contrast, the correlation between R-sided INVOS and Euroscore was negative and significant (r = -0.315, p < 0.001, Figure 4).

### Comparison of vascular vs. cardiac surgery patients

Carotid and cardiac surgery patients would be expected to have similarities, because risk factors for vascular and coronary artery disease are overlapping. Differences and similarities between these patients group are presented in Table 1, which shows that, compared to cardiac surgery patients, carotid surgery patients are older (71.05 ± 8.69 vs. 65.72 ± 11.04, P = 0.001), and have a much higher frequency of stroke (15 of 57, vs. 8 of 100, P = 0.002). In contrast, cardiac surgery patients have a significantly higher frequency of high cholesterol (53 of 100, vs. 14 of 57, P = 0.001) and hypertension (81 of 100 vs. 29 of 57, P = 0.0001), whereas the frequency of diabetes mellitus, smoking and male sex do not differ between groups. With regards to baseline INVOS values, carotid surgery patients have significantly higher baseline INVOS values on the left side (66.81 ± 8.17 vs. 63.25 ± 7.28, P = 0.007) and on the right side (65.91 ± 8.06 vs. 62.25 ± 8.04, P = 0.007). This consistent difference, with carotid surgery patients having significantly higher baseline INVOS values compared to cardiac surgery patients is also obvious when looking at percentiles: the lowest 5% of baseline INVOS values on the left/right side were 51/50 in carotid, vs. 52/46 in cardiac surgery patients, whereas the lowest 10% baseline values were 57/54 in carotid vs. 54/52 in cardiac surgery, and the lowest 20% of INVOS values were 60/59 in carotid surgery vs. 56/56 in cardiac surgery.

## Discussion

NIRS is a relatively new tissue oxygenation monitoring technology, and its use for monitoring brain oxygenation with INVOS may be a useful tool in an attempt to improve outcomes in carotid and cardiac surgery. Published data suggest that significant intraoperative reduction of INVOS values correlates with adverse outcomes (cognitive dysfunction, hospital length of stay), and preliminary data suggest that prompt interventions in episodes of reduced INVOS values may contribute to improved outcomes. However, in order to better understand the role of INVOS brain tissue oxygenation monitoring in clinical practice, more data are needed to establish baseline values and identify factors influencing INVOS measurement in different patient populations. Relevant data have already been published: baseline INVOS values in cardiac surgery were 58.6% ± 10.2% in the Yao study [7], and transient cerebral ischemia during carotid or cardiac surgery seemed to correlate with adverse neurologic outcomes. Our small study is an attempt to evaluate factors that could influence baseline INVOS values in patients undergoing cardiac or carotid artery surgery, and establish baseline reference values for Greek patients, a population where smoking is very common, and preventive medical care is inconsistent. Compared to the Yao study, baseline INVOS values in our study are higher in both carotid (66.81 ± 8.17) and cardiac surgery patients (63.25 ± 7.28), and the variance of baseline values in our population is smaller (as evidenced by smaller SD), perhaps due to greater homogeneity of our patient sample. Our results provide some insight on demographic and clinical factors that seem to influence baseline INVOS values, and identification of such factors may help us better assess the importance of deviations of intraoperative INVOS readings from baseline values.

## Conclusions

Our data suggest that, compared to cardiac surgery, carotid endarterectomy patients are older and have higher baseline INVOS values and greater stroke frequency. In contrast, cardiac surgery patients have higher frequency of high cholesterol and hypertension, whereas the two groups do not differ with regards to smoking and diabetes mellitus. High cholesterol and diabetes are associated with lower baseline INVOS values in carotid surgery patients. Right sided baseline INVOS values are strongly correlated with left sided INVOS values in both patient groups. Our data also suggest that baseline INVOS values in Greek patients undergoing carotid or cardiac surgery are higher and more homogeneous compared to patients in western European and North American studies.

As this is an observational study, and there was no intervention in response to observed INVOS values, our data cannot support any conclusions regarding intraoperative management of these patients. However, this prospective observational study provides some direction for future research on factors that may influence baseline and intraoperative INVOS values, but our patient number is relatively small, and does not allow for definite conclusions. Data from larger prospective studies are needed to evaluate the validity of our findings.

## Abbreviations

CABG: Coronary Artery Bypass Grafting; CAD: Coronary Artery Disease; CEA: Carotid Endarterectomy; CVA: Cerebrovascular Accident; DM: Diabetes Mellitus; HTN: Hypertension; ICA: Internal Carotid Artery; ICU: Intensive Care Unit; INVOS: IN Vivo Optical Spectroscopy; LOS: Length of Stay; LVEF: Left Ventricular Ejection Fraction; MAP: Mean Arterial Pressure; MI: Myocardial Infarction; NIRS: Near-Infrared Spectroscopy; POCD: Postoperative Cognitive Dysfunction; RCT: Randomized Controlled Trial; rSO2: Regional Tissue Oxygen Saturation; SD: Standard Deviation

## Competing interests

This work was supported solely by department funds. All authors declare that they have no competing interests to disclose.

## Authors' contributions

NB participated in patient care and collected data, MK analyzed data, wrote, revised and submitted manuscript, SS did all cardiac surgery operations, MM did all vascular surgery operations, GP designed and directed the study and revised the manuscript. All authors have read and approved the final manuscript.

## References

[B1] Hammon JW Jr Stump DA Kon ND Cordell AR Hudspeth AS Oaks TE Brooker RF Rogers AT Hilbawi R Coker LH Troost BT Risk factors and solutions for the development of neurobehavioral changes after coronary artery bypass grafting Ann Thorac Surg 1997 63 1613 1618 10.1016/S0003-4975(97)00261-0 9205158

[B2] Roach GW Kanchuger M Mangano CM Newman M Nussmeier N Wolman R Aggarwal A Marschall K Graham SH Ley C Adverse cerebral outcomes after coronary bypass surgery. Multicenter Study of Perioperative Ischemia Research Group and the Ischemia Research and Education Foundation Investigators N Engl J Med 1996 335 1857 1863 10.1056/NEJM199612193352501 8948560

[B3] Slater JP Guarino T Stack J Vinod K Bustami RT Brown JM III Rodriguez AL Magovern CJ Zaubler T Freundlich K Parr GV Cerebral oxygen desaturation predicts cognitive decline and longer hospital stay after cardiac surgery Ann Thorac Surg 2009 87 36 44 10.1016/j.athoracsur.2008.08.070 19101265

[B4] Newman MF Kirchner JL Phillips-Bute B Gaver V Grocott H Jones RH Mark DB Reves JG Blumenthal JA Longitudinal assessment of neurocognitive function after coronary-artery bypass surgery N Engl J Med 2001 344 395 402 10.1056/NEJM200102083440601 11172175

[B5] Casati A Spreafico E Putzu M Fanelli G New technology for noninvasive brain monitoring: continuous cerebral oximetry Minerva Anestesiol 2006 72 605 625 16865080

[B6] Botes K Le Roux DA Van MJ Cerebral monitoring during carotid endarterectomy--a comparison between electroencephalography, transcranial cerebral oximetry and carotid stump pressure S Afr J Surg 2007 45 43 46 17674560

[B7] Yao FS Tseng CC Ho CY Levin SK Illner P Cerebral oxygen desaturation is associated with early postoperative neuropsychological dysfunction in patients undergoing cardiac surgery J Cardiothorac Vasc Anesth 2004 18 552 558 10.1053/j.jvca.2004.07.007 15578464

[B8] Monk TG Weldon BC Garvan CW Dede DE Aa MT van der Heilman KM Gravenstein JS Predictors of cognitive dysfunction after major noncardiac surgery Anesthesiology 2008 108 18 30 10.1097/01.anes.0000296071.19434.1e 18156878

[B9] Murkin JM Adams SJ Novick RJ Quantz M Bainbridge D Iglesias I Cleland A Schaefer B Irwin B Fox S Monitoring brain oxygen saturation during coronary bypass surgery: a randomized, prospective study Anesth Analg 2007 104 51 58 10.1213/01.ane.0000246814.29362.f4 17179242

[B10] Edmonds HL Jr Pro: all cardiac surgical patients should have intraoperative cerebral oxygenation monitoring J Cardiothorac Vasc Anesth 2006 20 445 449 10.1053/j.jvca.2006.03.003 16750751

[B11] Beese U Langer H Lang W Dinkel M Comparison of near-infrared spectroscopy and somatosensory evoked potentials for the detection of cerebral ischemia during carotid endarterectomy Stroke 1998 29 2032 2037 9756577 10.1161/01.str.29.10.2032

[B12] Lee TS Hines GL Feuerman M Significant correlation between cerebral oximetry and carotid stump pressure during carotid endarterectomy Ann Vasc Surg 2008 22 58 62 10.1016/j.avsg.2007.07.022 18023554

[B13] Edmonds HL Jr Ganzel BL Austin EH III Cerebral oximetry for cardiac and vascular surgery Semin Cardiothorac Vasc Anesth 2004 8 147 166 10.1177/108925320400800201 15248000

[B14] Samra SK Dy EA Welch K Dorje P Zelenock GB Stanley JC Evaluation of a cerebral oximeter as a monitor of cerebral ischemia during carotid endarterectomy Anesthesiology 2000 93 964 970 10.1097/00000542-200010000-00015 11020747

[B15] Edmonds HL Jr Protective effect of neuromonitoring during cardiac surgery Ann N Y Acad Sci 2005 1053 12 19 10.1196/annals.1344.002 16179501

[B16] Jensen BO Rasmussen LS Steinbruchel DA Cognitive outcomes in elderly high-risk patients 1 year after off-pump versus on-pump coronary artery bypass grafting. A randomized trial Eur J Cardiothorac Surg 2008 34 1016 1021 10.1016/j.ejcts.2008.07.053 18778948

[B17] Murkin JM Postoperative cognitive dysfunction: aprotinin, bleeding and cognitive testing Can J Anaesth 2004 51 957 962 10.1007/BF03018479 15574542

[B18] Tan ST Cerebral oximetry in cardiac surgery Hong Kong Med J 2008 14 220 225 18525092

